# Medical student opinions on character development in medical education: a national survey

**DOI:** 10.1186/s13104-015-1434-z

**Published:** 2015-09-18

**Authors:** George B. Carey, Farr A. Curlin, John D. Yoon

**Affiliations:** Department of Medicine, The University of Chicago, Chicago, IL 60637 USA; Trent Center for Bioethics, Humanities & History of Medicine, Duke University, Durham, NC 27710 USA; Department of Medicine, MacLean Center for Clinical Medical Ethics, The University of Chicago, 5841 South Maryland Avenue, MC 5000, Chicago, IL 60637 USA

**Keywords:** Professionalism, Ethics, Religion, Medical education, Character development, Character feedback

## Abstract

**Background:**

Recently United States (US) medical schools have implemented curricular reforms to address issues of character in medical education. Very few studies have examined students’ opinions about the importance of character development in medical school. This cross-sectional study assessed US medical students’ opinions regarding character-focused education and their experiences receiving character feedback from educators. We mailed a questionnaire to 960 third year medical students from 24 medical schools. Respondents received a second questionnaire during their fourth year. Students answered three items that assessed their opinions regarding character development in medical education. They also indicated the frequency of positive/negative feedback regarding their character traits. We also tested associations between these opinions and various demographic, religious and spiritual characteristics. We used the χ^2^ test to examine bivariate associations between each demographic/religious characteristic and students’ opinions on character development or feedback.

**Results:**

Excluding 41 ineligible respondents, the adjusted response rate for the first questionnaire was 61 % (n = 564/919) and 84 % (n = 474/564) for the follow-up questionnaire. Twenty-eight percent of students agreed that one could be a good physician without being a good person; 39 % agreed that educators should focus on science instead of students’ characters; 72 % agreed that it was educators’ responsibility to train students to have good character; 1 % of students reported no positive feedback from faculty regarding character traits; 50 % reported no negative feedback.

**Conclusions:**

US students in clinical clerkships receive predominately positive feedback from educators regarding character traits. A majority of medical students, regardless of demographic and religious characteristics, are receptive to the role of character development in medical education. This finding suggests that character-based approaches toward ethics and professionalism training may find renewed receptivity among medical students despite recent “professionalism movement” fatigue.

## Background

Over the past few decades, medical schools throughout the United States (US) have implemented reforms to attend to character development in their ethics and professionalism curricula. These reforms have varied considerably across institutions, reflecting ongoing debates about various goals of ethics and professionalism training [1]. Historically, positions on the proper goals of ethics and professionalism curricula have fallen into two broad categories: (1) such training should equip students with a “skill set” for resolving ethical or professional issues, or (2) such training should produce “virtuous” physicians through character development [2–7]. With respect to the latter category, some prominent medical educators have called on academic medicine to renew a character or virtue-based approach toward medical education [7–9].

More recently, some have proposed curricular exercises that are designed to fulfill both of the above goals [8]. In the years since Hafferty first drew attention to the hidden curriculum, much attention has also been paid to the impact of the clinical years, role modeling, and mentorship on student character and moral development [10]. However, while educators acknowledge the role of positive character development in medical education, their practices of giving character feedback are highly variable. Moreover, a substantial body of literature has arisen in response to the recent “professionalism movement,” addressing conceptual debates and examining current practices, yet until now that literature has focused largely on the perspective of educators [2], though some work has been done from the perspective of patients [11, 12]. Medical students, for instance, anecdotally report a growing sense of “professionalism movement” fatigue due to the unprofessional conduct of their medical educators and the unfavorable learning environments of their institutional cultures [13, 14]. However, relatively little is known on a national level about US students’ opinions and experiences regarding their experience with character-based approaches in ethics and professionalism training. Outside of small, single-institution studies and anecdotal reports [13–19], little is known about students’ personal experiences of receiving character feedback from faculty [13–15, 20]. Nor have many studies examined students’ opinions about the theoretical importance of the character development in the process of medical training.

In this study, we used data from a national survey to assess US medical students’ opinions regarding character-focused medical education. We also inquired about the frequency of their experiences receiving feedback on character traits from medical educators. Because most religious traditions emphasize the importance of forming moral character over time, we also tested the hypothesis that more religious/spiritual students would be more supportive of character development in medical education than their less religious/spiritual counterparts.

## Methods

We performed this investigation as part of a larger study, the Project on the Good Physician, which seeks to understand the moral and professional development of American physicians during their medical training [21]. Pursuant with these goals, we developed and implemented a national pilot survey that addressed a wide range of topics relating to students’ opinions on and experiences with their medical training, including their opinions on the importance of character development in medicine. We examined a subset of the data collected from this survey for the purposes of this paper.

Following approval of the study by University of Chicago Social and Behavioral Sciences institutional review board, a cross-sectional survey (paper and online) was sent to 960 students from 24 medical schools throughout the United States in January 2011. These schools were chosen from the American Medical Association Physician Master File. From 133 allopathic medical schools, a nationally representative sample was selected using a systematic strategy based on probability proportional to school size and implicit stratification. This stratification was based on census region, public/private status, Social Mission ranking and whether the school had a Gold Humanism program. Students from each school were sorted by school year and randomized within their year. The first 40 third year medical students from the randomized list were selected to receive the survey. Written consent was waived by the IRB for this study, and those who did respond to the survey were understood as giving implied consent to have their responses included in our data collection. For students who completed the first round survey a follow-up survey was sent in September 2011 (when the third year students became fourth year students). Participants were paid an upfront incentive of $5 for their involvement in the first survey and an additional $10 for participation in the follow-up survey. Students who were not third year students at the time of the first survey were excluded from analysis (n = 41).

For this investigation, we examined responses to a subset of questions from both questionnaires. In the first questionnaire, students were asked how often they had received positive and negative feedback from educators regarding character traits since the beginning of their clinical rotations. Response categories were “Never,” “Once or twice,” “A few times,” “Several times,” and “Numerous times.” To assess opinions on character development, students were asked in the second questionnaire whether they agreed or disagreed with the following three statements: (1) “One can still be a good physician even if one is not a very good person,” (2) “Medical educators are responsible for training medical students to have good character,” and (3) “Medical educators should focus on teaching the science of medicine rather than trying to shape students’ character.” Response categories were “Agree strongly,” “Agree somewhat,” “Disagree somewhat,” and “Disagree strongly.” Terms such as “good character” and “character traits” were not defined or explained in any way for respondents.

We also examined student demographic, educational, and religious/spiritual characteristics in order to determine predictors for responses to the above questions. Students indicated their religious characteristics in the second questionnaire. Organizational religiosity was determined by frequency of attendance at religious services (never, less than once a year, about once or twice a year, several times a year, about once a month, two to three times a month, nearly every week, every week, several times a week). Intrinsic religiosity was measured using an abbreviated version of the Hoge intrinsic religiosity scale []. To determine the importance of religion, students were asked to rate how important religion is to them (most important, very important, fairly important, not important, not applicable). Theological pluralism—the belief that no religion is uniquely and comprehensively true—was also assessed as adapted from a previous study [23]. In order to assess student spirituality, students were asked the extent to which they considered themselves to be spiritual (very, moderately, slightly, not at all), adapting a measure of spirituality from a previous study [24]. School social mission score was taken from a previous study [25]. We used the χ^2^ test to examine bivariate associations between each demographic/religious characteristic and students’ opinions on character development or feedback. Blank responses were omitted from the analysis of those items. Data were analyzed with Stata v.12. Case weights were included in our analyses to generate national estimates from our sample as described in a related study [26].

## Results

After excluding 41 ineligible respondents (students who were not in their third-year clinical clerkships at the time of the survey), the adjusted response rate for the first questionnaire was 61 % (n = 564/919) and 84 % (n = 474/564) for the follow-up questionnaire. Demographic data for respondents can be found in Table 1. As seen in Table 2, 28 % of third year medical students agreed (somewhat or strongly) with the statement, “One can still be a good physician even if one is not a very good person”; 72 % agreed with statement, “Medical educators are responsible for training students to have good character”; and 39 % agreed with statement, “Medical educators should focus on teaching the science of medicine rather than trying to shape students’ character.” As seen in Table 3, only 1 % of medical students reported never receiving any positive feedback from attending faculty regarding character traits, whereas 50 % (n = 278/561) reported never receiving negative feedback.

**Table 1:** Demographic characteristics for respondents to both questionnaires

	Respondents to questionnaire 1 (N = 564)	Respondents to questionnaire 2 (N = 474)
	N (%)	N (%)
Sex		
Male	306 (54)	260 (55)
Female	258 (46)	214 (45)
Race/Ethnicity		
White, non-Hispanic	329 (58)	291 (61)
Black, non-Hispanic	48 (9)	39 (8)
Asian	128 (23)	99 (21)
Hispanic/Latino	28 (5)	22 (5)
Other	31 (6)	23 (5)
Region		
Northeast	137 (24)	113 (24)
South	204 (36)	171 (36)
Midwest	147 (26)	128 (27)
West	76 (13)	62 (13)

Percentages may not sum to 100 due to rounding

**Table 2:** Response totals and frequencies for questions regarding character development (total respondents = 474)

One can still be a good physician even if one is not a very good person			Medical educators are responsible for training medical students to have good character			Medical educators should focus on teaching the science of medicine rather than trying to shape students’ character		
Response	N	%	Response	N	%	Response	N	%
Agree strongly	19	4	Agree strongly	83	17	Agree strongly	41	8
Agree somewhat	115	25	Agree somewhat	261	56	Agree somewhat	145	30
Disagree somewhat	211	44	Disagree somewhat	115	24	Disagree somewhat	223	48
Disagree strongly	128	26	Disagree strongly	15	3	Disagree strongly	63	14
Total	473		Total	474		Total	472	

All percentages account for case weights. Percentages may not sum to 100 due to rounding

**Table 3:** Response totals and frequencies for questions regarding educator feedback on character traits during clerkships (total respondents = 564)

Received *positive* feedback from attending faculty regarding character traits			Received *negative* feedback from attending faculty regarding character traits		
Response	N	%	Response	N	%
Never	6	1	Never	278	50
Once or twice	44	7	Once or twice	172	33
A few times	93	15	A few times	79	12
Several times	202	37	Several times	24	4
Numerous times	217	40	Numerous times	8	1
Total	562		Total	561	

All percentages account for case weights. Percentages may not sum to 100 due to rounding. Question stem for this survey item was: “Since the beginning of your clinical rotations, how many times have you experienced each of the following?”

We did not find any significant associations between students’ opinions on character development and their demographic characteristics (age, gender, race/ethnicity, region), their religious or spiritual characteristics, or the social mission score of their medical school. Females were significantly more likely to report never having received negative feedback on character traits (43 % of males vs. 57 % of females, p = 0.005, data not shown in tables). No other significant associations were found between frequency of negative feedback and the above demographic, religious, and school characteristics. The frequency of positive feedback was not significantly associated with the measured demographic, religious, or school characteristics, except that it varied somewhat by geographic region (31 % of students in the Northeast had experienced numerous instances of positive feedback vs. 35 % in the South, 47 % in the Midwest, 53 % in the West, p = 0.02, χ^2^, data not shown in tables).

## Discussion

Most US medical students believe it is necessary to have good character in order to be a good doctor, and the majority seems to endorse the idea that medical educators should seek to train students to have good character. Students’ opinions did not vary by any measured student or school-level characteristics. Taken together, these findings suggest that a majority of medical students, regardless of demographic and religious characteristics, are receptive to character-based medical education. Character-based medical education is also supported by studies that suggest that patients themselves expect their physicians to be exhibiting ideal physician behaviors and positive character traits more related to the interpersonal quality than to the technical quality of their care [11, 12]. Student support for such education also seems to align with the larger goals of prominent medical governing and educational bodies. For example, the Association of American Medical Colleges (AAMC), Accreditation Council of Graduate Medical Education (ACGME), and American Board of Internal Medicine (ABIM) have continually reaffirmed the value of professionalism and have called for it to be a part of medical education [27–29]. Furthermore, the recent Carnegie Foundation’s Report on the centennial of the Flexner Report emphasizes forming “professional identity” in student physicians [30]. This language of fostering professional identity rather than that of merely encouraging professional behaviors reflects a broader trend toward addressing character education in substantive ways during medical training. The success of such efforts is largely unknown.

We should also note that professionalism education, and character development in particular, has met some criticism among medical students. Indeed, medical educators’ attempts to inculcate professionalism often meet “professionalism movement” fatigue among students [13, 14]. In their recent essay, Leo and Eagen, themselves medical students at the time of their writing, claim that students are affronted by faculty attempts to criticize or comment on perceived defects of character, especially when they witness unprofessional behavior among their own faculty [14]. Medical educators such as Daniel Sulmasy, however, have noted that moral development is a dynamic process during medical school, leading Sulmasy to ask whether medical schools can be transformed into “schools for virtue” [5]. He writes, “The cynics will contend that virtue cannot be taught, that students come to us already morally packaged and incapable of change. Against this…[data] show that students can, and in fact do, change. Unfortunately, this change is in the wrong direction” [5]. Indeed, students have been found to experience less moral progression than peers in other professions over the same period of education, and some have been found to undergo moral regression [31, 32]. Significant character development to counteract moral regression can occur in contexts outside of medical training. Our data, however, demonstrate that students believe medical school is an appropriate setting for efforts aiming at character development. It may be that students’ anecdotal resistance to educators’ efforts at character development is not necessarily a repudiation of the ongoing need for character development in medical education, but more of a protest of such efforts being done without equal attention to faculty development and accountability in these same areas [13, 14].

One practical question our study raises is whether character development can be effective when students appear to receive far more positive feedback than negative feedback regarding character traits. One obvious possible explanation for our findings is that the majority of medical students display good moral character, thereby earning much more praise than criticism from their faculty. It may also be, however, that educators tend to avoid giving negative feedback about student characters, because they are uncertain about how to do so effectively or they are uncomfortable doing so for other reasons [20]. Branch suggests that educators are at times afraid that criticizing trainee character may compromise the working relationships of the medical team [31]. Of note, our study also found that females were more likely to report never having received negative feedback on their character traits, which may indicate gender differences in how educators approach character education in medical training, or may indicate that female students in our study were more reluctant to report negative feedback. One study found that, in most cases, faculty did not respond to morally problematic behavior in residents and medical students, even when the faculty observed that behavior. When they did respond, they tended not to refer to the moral dimensions of unprofessional behavior [20]. Especially when viewed alongside this study, our data may indicate that “red flag” areas of character development are routinely neglected during medical training.

Our findings seem to support the recommendations made by Charles Bryan, who has proposed curricular reforms to achieve both goals of character development and ethics skill set development [8]. Drawing from recent work in positive psychology that suggest that character strengths and virtues can be assessed and taught, Bryan recommends a four-step method of reflective practice that integrate virtues and ethical principles in a longitudinal model of curricular reform. Recent faculty development initiatives to teach humanistic behavior among educators appear to show early promise, as do programs like the Gold Humanism Honor Society, which aim to impact institutional cultures as a whole [33–35]. Others have put forth a “moral intuitionist model of virtuous caring” [7] in which character development is best accomplished by tuning-up moral intuitions, amplifying moral emotions related to intuitions, and strengthening moral virtues, more than by “learning” explicit ethical rules or principles. However, a robust empirical agenda for a virtue-based medical education is still needed to better assess contemporary efforts to build character in American medical schools [7]. Character development of this sort has tended to emphasize the use of narrative [36], the creation of a rich community of learners [37], and a recovery of the longitudinal “apprenticeship” model of education in which lives of service are integrated into learning communities that connect “lived experiences of mentors and learners with an interdisciplinary set of didactic materials” [38].

There are several limitations to our study. First, medical students with greater interest in ethics and character may have been more likely to respond, leading us to overestimate student support for character development in medical school. Second, there is the possibility of recall bias, though these students were asked about their experiences receiving feedback from January to April which would have been right in the midst of their clinical clerkships. Nevertheless, it is possible that their experiences would have been different had they been asked to report on feedback received over the full duration of their clinical rotations. Third, we asked students for their opinion on the importance of character development in medical training, but we did not collect information about current implementation, nor did we provide a definition of “character” in our survey, leaving interpretation of this term up to the respondent. In our analyses, we also did not take into account their chosen specialty since students’ choice of specialty may also influence how they view the relevance of character development in one specialty over another. Moreover, while our data suggest that students support character development in principle, we cannot draw conclusions about whether or not they endorse current practices at their own medical schools.

## Conclusion

Limitations notwithstanding, this study demonstrates that US students in clinical clerkships receive predominately positive feedback from educators regarding character traits. Furthermore, our data indicate that US medical students appear to generally support the idea of character development as part of medical training, regardless of their demographic or religious characteristics. This finding suggests that character-based approaches toward ethics and professionalism training may find renewed receptivity among medical students despite recent “professionalism movement” fatigue.

## Abbreviations

US: United States
AAMC: Association of American Medical Colleges
ACGME: Accreditation Council of Graduate Medical Education
ABIM: American Board of Internal Medicine

## References

[1] Swick HM, Szenas P, Danoff D, Whitcomb ME (1999). Teaching professionalism in undergraduate medical education. JAMA.

[2] Eckles RE, Meslin EM, Gaffney M, Helft PR (2005). Medical ethics education: where are we? Where should we be going? A review. Acad Med.

[3] Kinghorn WA (2010). Medical education as moral formation: an Aristotelian account of medical professionalsim. Perspect Biol Med.

[4] Miles SH, Lane LW, Bickel J, Walker RM, Cassel CK (1989). Medical ethics education: coming of age. Acad Med.

[5] Sulmasy DP (2000). Should medical schools be schools for virtue?. J Gen Intern Med.

[6] Wong JG, Cheung EP (2003). Ethics assessment in medical students. Med Teach.

[7] Leffel GM, Oakes Mueller RA, Curlin FA, Yoon JD (2014). Relevance of the rationalist-intuitionist debate for ethics and professionalism in medical education. Adv Health Sci Educ.

[8] Bryan CS, Babelay AM (2009). Building character: a model for reflective practice. Acad Med.

[9] Coulehan J, Williams PC (2001). Vanquishing virtue: the impact of medical education. Acad Med.

[10] Hafferty FW, Franks R (1994). The hidden curriculum, ethics teaching, and the structure of medical education. Acad Med.

[11] Bendapudi NM, Berry LL, Frey KA, Parish JT, Rayburn WL (2006). Patients’ perspectives on ideal physician behaviors. Mayo Clin Proc.

[12] Fung CH, Elliott MN, Hays RD, Kahn KL, Kanouse DE, McGlynn EA (2005). Patients’ preferences for technical versus interpersonal quality when selecting a primary care physician. Health Serv Res.

[13] Brainard AH, Brislen HC (2007). Viewpoint: learning professionalism: a view from the trenches. Acad Med.

[14] Leo T, Eagen K (2008). Professionalism education: the medical student response. Perspect Biol Med.

[15] Terndrup C (2013). A student’s perspective on medical ethics education. J Relig Health.

[16] Baernstein A, Oelschlager AMEA, Chang TA, Wenrich MD (2009). Learning professionalism: perspectives of preclinical medical students. Acad Med.

[17] Lehrmann JA, Hoop J, Hammond KG, Roberts LW (2009). Medical students’ affirmation of ethics education. Acad Psychiatry.

[18] Roberts LW, Hammond KAG, Geppert CM, Warner TD (2004). The positive role of professionalism and ethics training in medical education: a comparison of medical student and resident perspectives. Acad Psychiatry.

[19] Shelp EE, Russell ML, Grose NP (1981). Students’ attitudes to ethics in the medical school curriculum. J Med Ethics.

[20] Burack JH, Irby DM, Carline JD, Root RK, Larson EB (1999). Teaching compassion and respect. J Gen Intern Med.

[21] Program on Medicine and Religion. Project on the Good Physician. 2015. http://pmr.uchicago.edu/projects/research/good-physician. Accessed 14 Sept 2015.

[22] Hoge R (1972). A validated intrinsic religious motivation scale. J Sci Study Relig..

[23] Yoon JD, Rasinski KA, Curlin FA (2010). Moral controversy, directive counsel, and the doctor’s role: findings from a national survey of obstetrician–gynecologists. Acad Med.

[24] Curlin FA, Chin MH, Sellergren SA, Roach CJ, Lantos JD (2006). The association of physicians’ religious characteristics with their attitudes and self-reported behaviors regarding religion and spirituality in the clinical encounter. Med Care.

[25] Mullan F, Chen C, Petterson S, Kolsky G, Spagnola M (2010). The social mission of medical education: ranking the schools. Ann Intern Med.

[26] Cook AF, Arora VM, Rasinski KA, Curlin FA, Yoon JD (2014). The prevalence of medical student mistreatment and its association with burnout. Acad Med.

[27] Accreditation Council on Graduate Medical Education (ACGME) Outcome Project. Advancing education in medical professionalism. 2004. http://www.usahealthsystem.com/workfiles/com_docs/gme/2011%20Links/Professionalism%20-%20Faculty%20Dev..pdf. Accessed 10 Feb 2014.

[28] ABIM Foundation, American Board of Internal Medicine (2002). Medical professionalism in the new millennium: a physician charter. Ann Intern Med.

[29] Association of American Medical Colleges. Assessment of professionalism project. 2014. https://www.aamc.org/download/77168/data/professionalism.pdf. Accessed 10 Feb 2014.

[30] Irby DM, Cooke M, O’Brien BC (2010). Calls for reform of medical education by the Carnegie Foundation for the Advancement of Teaching: 1910 and 2010. Acad Med.

[31] Branch WT (2000). Supporting the moral development of medical students. J Gen Intern Med.

[32] Feudtner C, Christakis DA, Christakis NA (1994). Do clinical clerks suffer ethical erosion? Students’ perceptions of their ethical environment and personal development. Acad Med.

[33] Branch WT, Frankel R, Gracey CF, Haidet PM, Weissmann PF, Cantey P (2009). A good clinician and a caring person: longitudinal faculty development and the enhancement of the human dimensions of care. Acad Med.

[34] Gold Humanism Honor Society. 2013. http://www.humanism-in-medicine.org/index.php/programs_grants/gold_foundation_programs/gold_humanism_honor_society. Accessed 10 Feb 2014.

[35] Higgins S, Bernstein L, Manning K, Schneider J, Kho A, Brownfield E (2011). Through the looking glass: how reflective learning influences the development of young faculty members. Teach Learn Med.

[36] Coulehan J (2005). Viewpoint: today’s professionalism: engaging the mind but not the heart. Acad Med.

[37] Remen RN, Rabow MW (2005). The healer’s art: professionalism, service and mission. Med Educ.

[38] Daaleman TP, Kinghorn WA, Newton WP, Meador KG (2011). Rethinking professionalism in medical education through formation. Fam Med.

